# Timing of the Departure of Ocean Biogeochemical Cycles from the Preindustrial State

**DOI:** 10.1371/journal.pone.0109820

**Published:** 2014-11-11

**Authors:** James R. Christian

**Affiliations:** 1 Canadian Centre for Climate Modelling and Analysis, Victoria, B.C., Canada; 2 Fisheries and Oceans Canada, Institute of Ocean Sciences, Sidney, BC, Canada; Centro de Investigacion Cientifica y Educacion Superior de Ensenada, Mexico

## Abstract

Changes in ocean chemistry and climate induced by anthropogenic CO_2_ affect a broad range of ocean biological and biogeochemical processes; these changes are already well underway. Direct effects of CO_2_ (e.g. on pH) are prominent among these, but climate model simulations with historical greenhouse gas forcing suggest that physical and biological processes only indirectly forced by CO_2_ (via the effect of atmospheric CO_2_ on climate) begin to show anthropogenically-induced trends as early as the 1920s. Dates of emergence of a number of representative ocean fields from the envelope of natural variability are calculated for global means and for spatial ‘fingerprints’ over a number of geographic regions. Emergence dates are consistent among these methods and insensitive to the exact choice of regions, but are generally earlier with more spatial information included. Emergence dates calculated for individual sampling stations are more variable and generally later, but means across stations are generally consistent with global emergence dates. The last sign reversal of linear trends calculated for periods of 20 or 30 years also functions as a diagnostic of emergence, and is generally consistent with other measures. The last sign reversal among 20 year trends is found to be a conservative measure (biased towards later emergence), while for 30 year trends it is found to have an early emergence bias, relative to emergence dates calculated by departure from the preindustrial mean. These results are largely independent of emission scenario, but the latest-emerging fields show a response to mitigation. A significant anthropogenic component of ocean variability has been present throughout the modern era of ocean observation.

## Introduction

The direct effect of anthropogenic CO_2_ on ocean chemistry already exceeds the range of natural variability in many locations [18]. However, many aspects of ocean biogeochemistry are forced only indirectly by CO_2_, via the effect of atmospheric CO_2_ on climate. Detecting an anthropogenic climate change signal in ocean biogeochemical data is difficult due to short data records and high natural variability [10], [13], [24]. Trends are not monotonic, and even strong anthropogenic forcing is subject to modulation by a variety of physical and biogeochemical processes [14]. In addition, the effects of climate warming are complex and competing processes can offset each other. For example, primary production will tend to increase with increasing temperature, but the same increases in temperature cause increasing stratification that limits the supply of nutrients to the surface ocean [37]. In addition, some fields have opposing trends in different regions [23].

Variability in climate can be divided into forced and unforced components. In model simulations the unforced component is referred to as the model's internal variability, or natural variability. Models never reproduce the timing of natural variability exactly, but a good model will reproduce the statistical characteristics [38]. A “white noise” spectrum implies approximately equal variability across the spectrum of frequencies resolved, while a “red noise” spectrum implies greater variance at lower frequencies (i.e., significant autocorrelation in time). Climate variability is generally considered to have a “red” spectrum [19], [25], but modern data records are too short for complete characterization and it is therefore difficult to know with confidence whether the models' 'natural' variability is too weak (or too strong). Nonetheless we know that climate models can accurately reproduce important aspects of observed climate variability [17], [40].

There is a relatively well-established, although certainly not uniform, methodology for detection of anthropogenic climate change and attribution of those changes to specific forcing factors [6], [9], [20], [34]. In this literature only a handful of papers to date have dealt with ocean biogeochemistry [1], [8], [18], [24]. Detection normally refers to a demonstration that observed variability in the climate system exceeds the range expected from natural variability at some specified (e.g., 5%) level of significance [5], [21]. Detection period refers to the length of a data record required to unequivocally detect an anthropogenic signal, while detection time indicates the point in time at which the signal becomes detectable [35]. The latter is related to the “time of emergence”, or the point in time at which the anthropogenic signal emerges from the “historical envelope” of natural variability, but there are subtle differences between the two. In model simulations, detection of even extremely small signals is possible given a sufficiently large ensemble; so one could argue that the term should not be used at all for studies using only model simulations. Emergence implies that thereafter the signal remains consistently outside the envelope of natural variability as defined e.g., by some multiple of the standard deviation of the unforced control simulation [29], [31]. In this study I define emergence as the point at which the anthropogenic signal exceeds natural variability (estimated from a preindustrial control simulation) at some specified threshold, and remains continuously in excess of that threshold (except when reversals are clearly attributable to mitigation). Note that this definition differs from that of [29], who include both natural and anthropogenic forcing up to 2005 as part of the envelope of historical variability from which emergence is estimated. It is also important to note that as observing systems inevitably have incomplete coverage, the point of potential detection of an anthropogenic signal is always later than the point of emergence as defined here.

While this sort of analysis does not offer any immediate hope of unambiguously detecting an anthropogenic signal in the real world, it represents a useful measure of the 'true' point of departure from the preindustrial climate, which is surprisingly early in many cases. Having an estimate of the emergence time is useful when examining historical data records, few if any of which are entirely free of anthropogenic influence. It can also be useful to impacts research in that it provides information about the magnitude of the anthropogenic signal relative to the natural variability [9], [27].

Modern coupled climate/carbon cycle models provide a homogeneous data set with which to conduct such an experiment, which despite its shortcomings overcomes some of the problems of earlier studies. Some early detection studies were limited by the lack of extended control simulations with models identical to their forced runs, or their control runs were done with ocean-only models for which stochastic forcing had to be employed to generate internal variability [35]. The current data set includes multiple realizations of the forced simulations, and an extended control run (with an identical model) from which the preindustrial variability can be estimated. It can not be known with certainty that the model does not underestimate natural variability, but if such biases exist they are probably small [12].

## Methods

### Model description

The model used is the Canadian Earth System Model, which has been previously described by [3], [4], [12]. In the version used here (CanESM2.0), the atmosphere model is run at T63 spectral resolution, a 128×64 horizontal grid. The 256×192 ocean model, with six grid cells (2x, 3y) to each atmosphere grid cell, has a longitude resolution of approximately 1.4° and latitude resolution of approximately 0.94°. The ocean model has 40 vertical levels (increased from 29 in CanESM1, with all of the additional levels in the upper 300 m), with a resolution of 10–15 m in the upper 100 m. The ocean carbon cycle model is based on the Ocean Carbon Model Intercomparison Project II protocols [32] and couples the carbon cycle to an NPZD ecosystem model via a fixed C/N Redfield Ratio and a temperature-dependent rain ratio (ratio of inorganic to organic carbon in vertical flux at the base of the euphotic zone) [12], [41].

Simulations used here have specified atmospheric CO_2_ concentrations; runs with freely-varying atmospheric CO_2_ give similar results. Historical (1850–2005) forcing includes volcanic eruptions and solar variability. Greenhouse gas forcing after 2005 is provided by the Representative Concentration Pathways (RCPs) [30]. RCPs 8.5, 4.5, and 2.6 are often referred to as the “no mitigation”, “moderate mitigation”, and “strong mitigation” scenarios respectively; in RCP8.5 emissions continue to increase and atmospheric CO_2_ concentration exceeds 900 ppm by 2100 [4].

**Figure 1:**
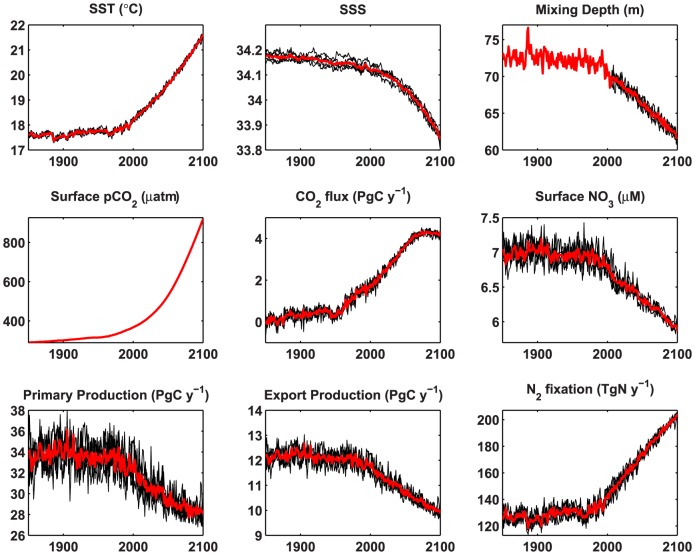
Global means or integrals of ocean surface fields from 1850–2100 under RCP8.5. Five ensemble members (thin black lines) are shown along with the ensemble mean (thick red line). Mixed layer depth has only a single realization for the historical run. Export is at 100 m. Primary production, export, CO_2_ flux and N_2_ fixation are global integrals; other fields are global mean surface values.

Nine data fields were considered: sea surface temperature and salinity, mixed layer depth (MLD), surface ocean pCO_2_ and air-sea CO_2_ flux, surface nitrate concentration, total primary production, organic export flux at 100 m, and dinitrogen fixation. All of these are standard (2D, monthly) data fields of the 5^th^ Coupled Model Intercomparison Project [39]; all data are in the public domain. The choices are somewhat arbitrary, but cover a range of fields commonly measured by oceanographers and used to diagnose the performance of ocean biogeochemical models. MLD was excluded from some analyses because in the historical simulation it was available only for a single realization (see below Figure 1).

### Statistics

For global and station means, emergence from the envelope of natural variability was defined as the first year the mean of an ensemble of five realizations of the forced (historical, RCP) run differed from the mean of the unforced preindustrial (1850 greenhouse gas concentrations) control run by n standard deviations (nσ) of the interannual variability of the control run, and remained in excess of nσ thereafter, with n = 3 for global metrics [31] and n = 2 for individual locations. The different criteria for global means and specific locations are arbitrary but yield consistent results across the range of stations considered; for individual locations a 2σ threshold was applied, because in many cases emergence would not occur by 2100 with the more conservative 3σ threshold. The ensemble mean is used to isolate the anthropogenic signal by averaging out the effect of internal variability. Three hundred years of control run were used to calculate the standard deviation. Annual means were used in all cases. Trends were not corrected for drift in the control run, but drift is small as only the surface and euphotic zone are considered, and is also present in the forced runs. Drift will bias the method slightly towards later emergence because it increases the preindustrial variability. In some cases the fields emerged but later fell back within the nσ window under the “strong mitigation” scenario (RCP2.6); these were considered emergent if they remained outside the window for 50 years (in practice, 20–25 years would be sufficient as the longest any fields remained outside the window in an unforced control run was ∼15 years).

Spatial ‘fingerprints’ were calculated for the 14 ocean regions defined by Sarmiento et al. [36] (hereafter S02) and for zonal means of 10° latitude bands as in [31]; results are largely insensitive to which of these was used (see below Results). S02 divided the ocean up by both latitude (regions are bounded by 15° and 45° north and south and exclude areas north of 60°N) and basin, even in the Southern Ocean which was divided into several ‘sectors’. So if there are interbasin differences in trends there will be a small amount of information in this pattern that is not present in simple zonal means.

Prior to spatial averaging, data were normalized as
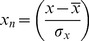
(1)


Where 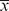 and 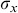 are the mean and standard deviation of all valid data from the preindustrial control run, except for dinitrogen fixation (DNF) where only data equatorward of 40° latitude were considered. The difference between the means for these regions for 2081–2100 of RCP8.5 and the means for 300 years of unforced control run was considered to be the 'fingerprint' of anthropogenic climate change. The projection of this fingerprint onto each year of the forced (historical + RCP) run, i.e.,
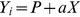
(2)where *X* is the anthropogenic fingerprint, *Y_i_* is a vector of regional means for the current year of the forced run and *P* is a vector of regional means for the long-term mean of the unforced control run, was calculated by linear least squares.
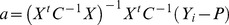
(3)where *C* is the covariance matrix for the control run. The same method was applied to (300) individual years (*P_i_*) of the control run in order to generate a distribution of values (*a_0_*) from which emergence can be determined. As for the global means, the year of emergence (YOE) was considered to be the year in which *a* exceeded three times the standard deviation of *a_0_* (*σ_a0_*) and remained consistently in excess of 3*σ_a0_* thereafter [31].

A 'combined' fingerprint experiment was conducted where the time series of regional means of all variables except pCO2, CO2 flux and mixing depth were superimposed, for a total of 92 variables/regions in the S02 case (sea surface nitrate concentration in the tropical Atlantic was excluded because its extremely low variance made the covariance matrix singular). pCO2 and CO2 flux were excluded because their much earlier emergence would dominate the result (pCO2 in particular has a much smaller preindustrial standard deviation than other fields and emerges very early); mixing depth was excluded because there was only a single realization of the historical run available. As noted above, high-latitude regions were excluded for DNF, because it only occurs where temperature exceeds 20°C.

### Ocean observing station network

Model simulations were sampled at ten ocean observing stations, eight of the nine used by Moore et al. [28] and Station KNOT in the northeast Pacific (Table 1). The Ross Sea station used by Moore et al. was replaced, because of its shallow depth and proximity to the ice shelf, with a more oceanic location in the South Atlantic (SATL). These stations were chosen to represent most major regions of the world ocean, excluding the Arctic and the marginal seas, and to include actual sampling stations where observations have been made. Four of the stations are located in the tropics and subtropics and three each in the Southern Ocean and the northern midlatitudes (Table 1). Northern midlatitude stations range in latitude from 44–50°N, and Southern Ocean stations from 51–62°S.

**Table 1:** Locations of stations at which the model simulations were sampled for local emergence.

Station	Latitude	Longitude
BATS	32°N	64°W
HOT	23°N	158°W
PAPA	50°N	145°W
KNOT	44°N	155°E
ARAB	16°N	62°E
EQPAC	0°	140°W
SATL	51°S	19°W
NABE	47°N	19°W
KERFIX	51°S	68°E
PLRFR	62°S	170°W

At each station, the YOE was determined in the same manner as for the global means, except that the criterion for emergence was set to 2σ instead of 3σ. In addition, 20 and 30 year linear trends were calculated for individual ensemble members to identify the point at which these become consistently positive or negative, termed the “Last Zero Crossing” (LZC). In this case individual ensemble members are used because the influence of natural variability must be preserved. For a LZC to be recorded for N year trends, the last sign reversal must occur at least N/2+10 years prior to the end of the run for at least 4 of 5 ensemble members; LZC is then averaged over the realizations in which a LZC was recorded.

## Results

### Global mean trends

Global mean trends in ocean physical and biogeochemical fields show substantial alteration in the 21st century under the no-mitigation scenario (Figure 1), and in many cases these trends are well underway by the end of the 20^th^ (which is scenario-independent). MLD declines by ∼3 m by 2000 and 10 m by 2100 (Figure 1). Export production and dinitrogen fixation show more or less monotonic trends that are well underway by 2000 (Figure 1). Ocean CO_2_ uptake continues to increase, but the rate of growth declines rapidly near the end of the 20th century (Figure 1). Some biogeochemical fields, such as primary production, have weak trends due to competing influences of e.g., temperature and stratification, as well as offsetting trends in different regions, so that the trend is relatively small compared to natural variability, at least initially (Figure 1).

**Figure 2:**
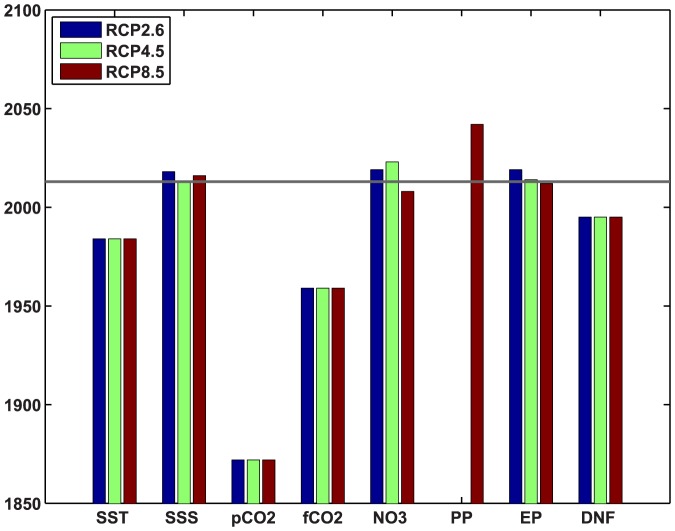
Year of emergence of global mean ocean surface fields from the range of natural variability for RCPs 2.6, 4.5, and 8.5. Emergence is defined as the year that the ensemble mean of 5 forced (historical + RCP) runs exceeds three times the standard deviation (3σ) of 300 years of unforced control run and remains in excess of 3σ thereafter. Horizontal grey line indicates beginning of 2013. SST  =  sea surface temperature; SSS  =  sea surface salinity; pCO2  =  surface ocean pCO_2_; fCO2  =  ocean-atmosphere CO_2_ flux; NO3  =  surface nitrate concentration; PP  =  primary production; EP  =  export production at 100 m; DNF  =  dinitrogen fixation.

The emergence of the global mean or integral values of the selected fields from the envelope of natural variability was tested by comparing ensemble means for historical + RCP2.6/4.5/8.5 simulations with the unforced control simulations, for each year of the simulation (1850–2100). Year of emergence was recorded as the first year that the ensemble mean differed from the preindustrial mean by at least 3 standard deviations of the control run and remained in excess of this threshold continuously thereafter. Surface ocean pCO_2_ emerges in the 1870s, but air-sea CO_2_ flux does not emerge until about a century later (Figure 2). Sea surface temperature (SST) emerges before sea surface salinity (SSS), as is expected for the global mean. SSS has offsetting trends in different regions, because the global surface net freshwater flux is close to zero, but anthropogenic warming increases the local flux in both net evaporative and net precipitation regions [15], [23]. Emergence for SST closely approximates the date at which detection of an anthropogenic contribution to ocean heat content change is deemed to have become statistically significant [9]. YOE for MLD was not determined but would be around 2005 (not shown).

Primary production does not emerge in RCPs 2.6 or 4.5, and even in RCP8.5 does not emerge until the 2040s. This is partly due to offsetting increasing and declining trends in different regions (see below sections 3.2 and 3.3). In RCP4.5 global mean primary production exceeds the 3σ threshold for about 10 years in the 2040s, but does not remain there because the overall trend is not monotonic under the mitigation scenarios (not shown). In RCP2.6, air-sea CO_2_ flux, surface nitrate concentration, and export production all fall back within the 3σ window after initially emerging from it the early 21^st^ century (not shown); in these cases the fields are recorded as emergent (Figure 2), because they remained outside the 3σ window continuously for>50 years. Note that emergence can occur slightly earlier in a lower emission scenario due to natural variability, which is reduced but not eliminated by using ensemble means (Figure 2). In fact, it is possible for emergence to occur after 2005 in one scenario while occurring before 2005 in the others, even though atmospheric CO_2_ is identical up to 2005.

### Detecting the spatial ‘fingerprint’ of anthropogenic change

**Figure 3:**
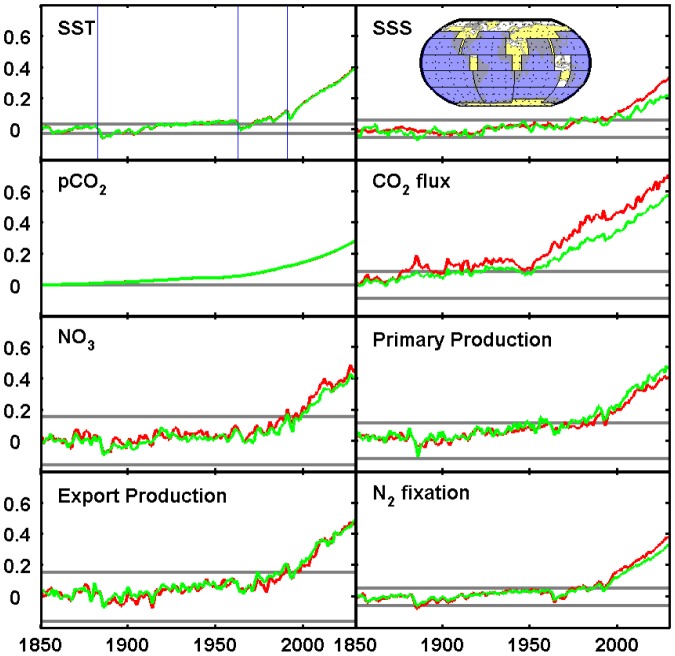
Contribution of 2081–2100 (RCP8.5) anthropogenic fingerprint to difference of current year from preindustrial, for individual fields. Fingerprint is based on areal means for the 14 ocean regions of Sarmiento et al. (2002) (S02, red) or global zonal means of 10° latitude bands (green). Vertical axes are normalized as shown in equation (1). Horizontal black lines are plus or minus three standard deviations of preindustrial values (3σ range is shown for S02 only; values are almost identical for the two methods). Vertical blue lines in first panel indicate eruptions of Krakatoa (1883), Agung (1963), and Pinatubo (1991). Inset map in second panel indicates S02 regions (blue rectangles); Pacific regions are single boxes spanning the basin.

Because some fields have offsetting increases and decreases in different regions [23], global means are not necessarily a good metric of whether the forced simulation has emerged from the envelope of variability of the unforced control. I have calculated a spatial ‘fingerprint’ of anthropogenic change by taking the difference between the mean for 2081–2100 of RCP8.5 and the mean of the preindustrial control run (equation 2). The projection of the anthropogenic fingerprint onto individual years of the transient run (*a* in equations 2 and 3) for individual data fields is shown in Figure 3 for two different choices of the averaging regions. The difference between the two is generally small and emergence from the 3σ window occurs by 2005 in all cases. Historical volcanic eruptions such as Krakatoa (1883), Agung (1963) and Pinatubo (1991) are visible in the trends for some fields (Figure 3). The eruption of Pinatubo occurs near the point of emergence for primary production, export production and dinitrogen fixation and delays emergence by 5–10 years (Figure 3).

Trends in the presence of the anthropogenic fingerprint do not in most cases differ much between the 14 regions of S02 or global zonal means for 10° latitude bands as in [31] (Figure 3). The variables that show the largest difference, i.e. the most sensitivity to basin-specific information, are sea-surface salinity and air-sea CO_2_ flux (Figure 3). There are large interbasin differences in evaporation and precipitation [11], so it is not surprising that the spatial ‘fingerprint' of anthropogenic warming for SSS has a larger component that is basin-specific than for most other fields. For CO_2_ flux the anthropogenic fingerprint shows enhanced net uptake (which may be reduced outgassing in outgassing regions) in the equatorial Pacific upwelling zone, the Gulf Stream and Kuroshio termination regions, the high-latitude North Atlantic and much of the Southern Ocean (not shown). However, there is a fair amount of variation among regions of the Southern Ocean, with the strongest enhancement in the Atlantic sector and a mosaic of positive and negative trends in the Pacific and Indian sectors. By recalculating the fingerprint for a reduced set of regions, with different basins being combined for specific latitude ranges, the latitudes in which basin-specific information is important can be identified (not shown). For CO_2_ flux about half of the total effect is in the Southern Ocean, with the balance in the tropics. For SSS the only region where there is sensitivity to basin-specific averaging is the subtropics (i.e., regions of net evaporation).

**Figure 4:**
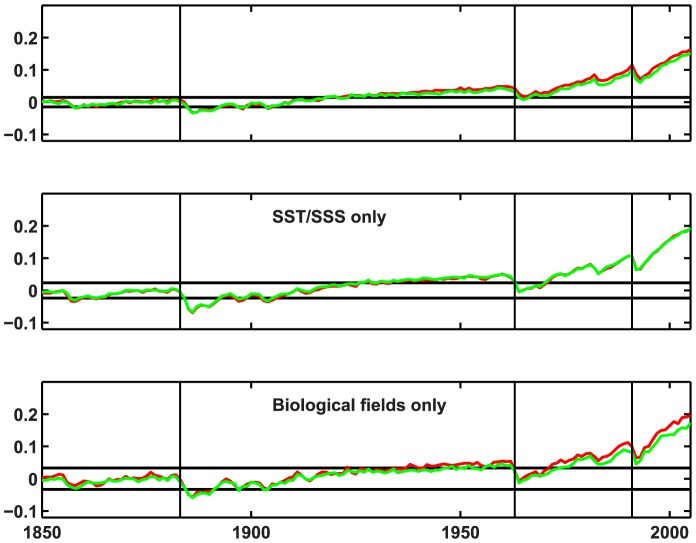
As Figure 3 but for combined fingerprint of all fields except pCO_2_, CO_2_ flux and mixing depth, for SST + SSS only, and for biological fields (surface nitrate concentration, primary production, export production and dinitrogen fixation) only. Red line indicates fingerprint based on the 14 ocean regions of S02; green line is for global zonal means of 10° latitude bands. Vertical black lines indicate eruptions of Krakatoa (1883), Agung (1963), and Pinatubo (1991).

The combined fingerprint of all non-carbon fields (except MLD) shows early emergence, and is also insensitive to the choice of averaging regions (Figure 4, Table 2). As with some of individual fields, volcanic eruptions can delay emergence. In this case the eruption of Agung (1963) delays emergence by nearly half a century in the zonal means case, whereas the S02 case is unaffected because it remains slightly outside the window following the eruption (Figure 4, Table 2). This illustrates how sensitive the exact date of emergence can be to the somewhat arbitrary criteria employed, but further serves to illustrate that an anthropogenic signal was present even in the first half of the twentieth century. The biological fields (surface nitrate concentration, primary production, export production and dinitrogen fixation) do not affect emergence time much compared to SST and SSS alone (Table 2), but this results in large part from the particular timing of the eruption of Agung (Figure 4). Inclusion of both sets of fields narrows the window of preindustrial variability substantially relative to either alone (Figure 4). Had the eruption not occurred when it did, the differences in emergence times among these three cases could be much larger.

**Table 2:** Year of emergence by estimation of single or multiple field anthropogenic fingerprint calculated for the 14 ocean regions of Sarmiento et al. (2002) or for global zonal means for 10° latitude bands, for two or three standard deviations of preindustrial values.

Regions	S02	S02	Zonal	Zonal
Emergence threshold	2σ	3σ	2σ	3σ
**Individual**				
SST	1972	1973	1972	1973
SSS	1976	1990	1997	1997
Surface pCO2	1859	1860	1859	1860
Air-sea CO2 flux	1877	1913	1918	1953
Surface [NO3]	1986	2000	1996	2000
Primary production	1969	1995	1973	1984
Export production	1988	1996	1981	1997
N2 fixation	1994	1995	1994	1995
**Combined**				
All, except pCO2 and CO2 flux	1919	1923	1967	1968
Biological fields	1971	1972	1971	1972
SST + SSS only	1971	1973	1973	1973

Combined fingerprint excludes pCO_2_ and CO_2_ flux; biological fields are surface nitrate concentration, primary production, export production and dinitrogen fixation.

### Local emergence and ocean observing networks

An increasing number of ocean time series data span several decades [7], [14]. But since these are largely localized observations, and interannual to interdecadal variability is also present in the data records, how can such data be used to detect a longer-term trend? And how clearly can such trends be associated with anthropogenic forcing? The following analysis of a network of 10 ocean observing stations explores these questions.

**Figure 5:**
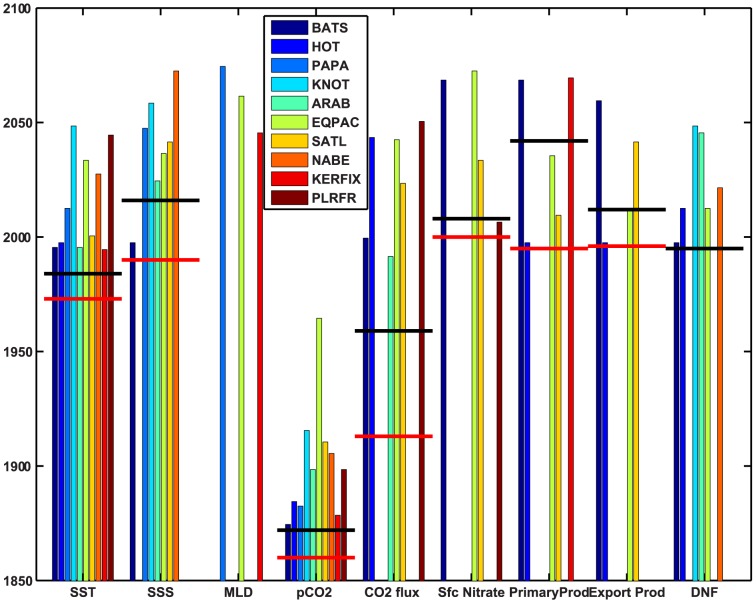
Year of emergence of mean ocean surface fields from the range of natural variability at 2σ for individual locations under RCP8.5 (see Table 1). Horizontal lines indicate YOE's for global means or spatial fingerprints from S02 (see Figures 2 and 3). Black indicates YOE for the global mean, red for the spatial fingerprint. For dinitrogen fixation red bars are not visible because YOE's are the same. No vertical bar indicates that the variable does not emerge by 2100 at that location.

Year of emergence at 2σ for the ten station means is shown in Figure 5. YOE's are generally later for individual stations than for the global means, and are quite variable among stations (Figure 5). However, the ranges for different variables are generally consistent with YOE's estimated for global means or spatial fingerprints except for CO_2_ flux (Figure 5). Surface ocean pCO_2_ emerges much earlier than other fields (prior to ∼1960), but the range among stations is almost 100 years. For all fields except pCO_2_ and SST, there are some stations at which emergence does not occur by 2100, and a few (MLD, surface nitrate, and export production) emerge at less than half of the stations (Figure 5).

Quasi-linear trends have been shown to occur over extended periods at ocean observing stations, particularly for carbon-related fields, for which the anthropogenic trend is large relative to the natural variability [7]. However, for other fields the time series are as yet too short for trends to be unambiguously associated with anthropogenic forcing. I have calculated 20 and 30 year linear trends from the model solution at the 10 observing stations in Table 1, and compared the time of emergence as estimated above with the time of the last sign reversal, or LZC.

The LZC for various fields and stations is shown in Table 3. The table shows the final year where the sign of the regression coefficient was opposite to the mean for the last 10 intervals. The LZC is not a statistically rigorous estimate of the time at which the anthropogenic trend becomes significant relative to natural variability, but it does give a rough indication (i.e., in the control climate the coefficients are equally often positive and negative), and it is shown below that its relation to the YOE is quite consistent. In quite a few cases sign reversals occur throughout the 20th century (blank entries in Table 3). In other cases the LZC occurs quite early, suggesting that the anthropogenic impact is present in the observations taken at these locations, which in most cases date from the 1980s or 1990s.

**Table 3:** Last year where 20 or 30 year trend had opposite sign to that recorded at the end of the 21st century.

	# years	BATS	HOT	PAPA	KNOT	ARAB	EQPAC	SATL	NABE	KERFIX	PLRFR
SST	20	1979	2027	2011	2041	1983	2058	2019	2049	1999	
	30	1968	1968	1974	1977	1967	1979	1968	2001	1957	2073
SSS	20	1980		2074	2080	2039	2059	2050	2060	2067	
	30	1958	2060	2038	2038	1997	2015	2021	2021	2064	
Mixing Depth	20			2070			2076	2077	2077		2075
	30	2073	2074	2049			2021	2056	2054		2070
Surface pCO_2_	20	1869	1931	1883	1963	1947	1972	1943	1936	1909	1942
	30	*	1869	*	1940	1873	1941	1885	1867	*	1853
Air-sea CO_2_ flux	20	2083		2062	2084	2044		2041	2078		2059
	30			2046		1993		1993	2057		2021
Surface [NO_3_]	20		2081		2082			2053		2074	
	30	2029			2081		2069	2036	2046	2073	
Primary production	20		2052		2082		2085	2051		2069	2079
	30	2030	1968		2072		2047	1976	2046	2047	
Export production	20		2069		2082	2074	2085	2080		2075	2079
	30	2019	1988		2081	2072	2059	2033	2044	2066	2075
N_2_ fixation	20	2001	2055			2083	2078				
	30	1968	1968			2071	2008				

No data indicates sign reversals continue up until the end of the 21st century, except for N_2_ fixation which only occurs at the stations where dates are listed.

* indicates that the trend was of a consistent sign from the outset. Mixing depth has only a single realization for the historical simulation but no values <2005 appear.

**Figure 6:**
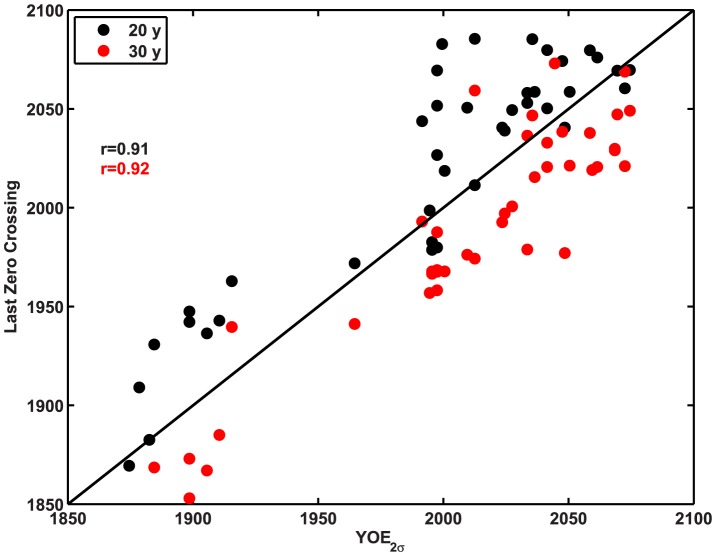
Last-zero-crossing for 20- and 30-year regression coefficients (Table 3) relative to YOE defined as in Figure 5, for all data fields at individual sampling stations where a last-zero-crossing was recorded.

Because in this experiment the YOE (based on the ensemble mean and preindustrial standard deviation) is known (Figure 5), one can determine whether the LZC is correlated with the YOE across different fields and locations and whether it tends to over- or underestimate the YOE. The LZC as an estimate of emergence time is strongly correlated with YOE for both 20 and 30 years trends, with r = 0.91 and 0.92 respectively (Figure 6). It provides consistently conservative estimates (exceeds the YOE) for 20 year trends, but not for 30 year trends, which tend to have an “early emergence” bias (Figure 6). This suggests that the proper trend length for which the LZC will approximate the YOE is ∼25 years, but the exact value is sensitive to the characteristics of the specific data sets employed (see Discussion).

## Discussion

A central result of this analysis is that if the model is a reasonable representation of the real world, most of the fields have already emerged from the preindustrial range (Figures 2 and 3, Table 2). As a result, the emission scenario does not matter much for the global YOE because it precedes the point where they start to diverge. However, for individual locations YOEs are generally later, sometimes much later (Figure 5) and therefore emergence may not occur, depending on the future trajectory of emissions. Global YOE is affected by mitigation only for primary production (Figure 2).

YOE is strongly affected by historical volcanic eruptions, and in some cases delayed by decades (Figures 3 and 4). The eruption of Mt Pinatubo (1991) delays emergence by about 10 years for several fields (Figure 3). Eruption of Agung in 1963 delayed emergence in the combined fingerprint experiment by more than 40 years in the zonal means case, while the S02 case is unaffected because it remains slightly outside the window following the eruption (Figure 4). Note that the downward trend at this time appears to begin prior to the eruption: this may result in part from natural variability, in part from errors in the volcanic aerosol data set, and in part from a small increase in volcanism globally in the years 1960–1963; this downward trend is present in multiple models (see e.g., [22]).

The spatial fingerprint analysis provides a measure of the importance of diverse observations for detection of anthropogenic effects. The analysis presented here does not deal with detection directly, because only model simulations are used, but it is likely that earlier YOE implies earlier detection assuming sufficient observations are available. Spatial fingerprints generate earlier YOE than the global means for all fields except DNF (Figure 5). In many cases basin-specific information provides little additional information above what is provided by global zonal means (Figure 3, Table 2). A combined fingerprint of all fields results in very early emergence (∼1920 were it not for the eruption of Agung in 1963). Inclusion of the biological fields has relatively little effect on time of emergence beyond what is available from SST and SSS alone, but does make the window smaller, i.e., the additional information increases confidence that the projection of the anthropogenic fingerprint on the preindustrial climate is close to zero (Figure 4). This is probably because the SST response to planetary heating is global and more or less instantaneous (disregarding modulation by natural variability), whereas impacts on ocean biology are more indirect, resulting from stratification that derives from surface heating. It is therefore unsurprising that fields like export production emerge later and have relatively little effect on the combined fingerprint YOE. DNF is (in the model) an approximately linear function of SST, so it has strong trends but contains little information beyond what is present in SST alone. Nonetheless, emergence of the combined fingerprint for biological fields alone is quite similar to the total fingerprint, so there is likely to be an anthropogenic influence on most modern ocean biogeochemistry measurements.

Simplifying assumptions such as a single plankton species with fixed elemental ratios limit variability of the modelled ocean ecosystem. Plankton models with multiple species are better able to reproduce changes in ecosystem structure that occur under different physical forcing regimes [2]. There is some evidence that changes in plankton elemental stoichiometry can also 'amplify' the biological response to relatively small changes in physical forcing [26]. These biases are present in both the control and the forced runs, but they bias the model towards a 'damped' biological response to changing physical forcing and thus induce a bias towards later emergence. Excluding DNF at higher latitudes also potentially biases the method towards later emergence because a small amount of DNF occurs in regions where it is absent in the control run (as SST begins to exceed 20°C), but these rates are very low (not shown).

The use of ensembles to average out internal variability in the transient runs gives a statistically robust estimate of emergence relative to the variability of the control run. The time of emergence in an individual realization would depend strongly on internal variability, because natural variability superimposed on an overall trend will tend to produce periods where the record is flat or has a weak counter-trend (similar to the recent “warming hiatus”, cf. [16]), followed by periods of rapid change. If there were no overall trend, the positive and negative trends would be symmetric (as in the unforced control run). But in the presence of a long term trend they are not, and there will be short periods with strong positive trends.

To fully understand the probability of medium-term trends occurring, one would ideally wish to know the frequency spectrum of natural variability. In the model, the trends are clearly forced by anthropogenic greenhouse gases; they do not occur in the unforced control run. But it is not known whether the model ocean underestimates (or overestimates, although that is less likely) natural variability [12], [25]. In the forced model runs, the probability of a trend occurring is inversely proportional to the length, i.e., 20 year countertrends appear much more often than 30 year ones (see Table 3). But in a stationary climate it is not always true that shorter countertrends appear more frequently; it depends on the frequency spectrum. The more ‘red’ the spectrum (i.e., the stronger the autocorrelation), the more likely it is that a longer period with a trend will occur. If the spectrum is sufficiently 'red', longer periods with a consistent trend can occur more frequently than short ones [19]. In the model this does not occur, and there is a clear association of longer trends with external forcing [16], but it is not possible to know with certainty whether the model's internal variability has a less ‘red’ spectrum than the real ocean. At some locations, such as BATS in the subtropical Atlantic, the model's variance spectrum is red, and longer periods with consistent trends occur more frequently than shorter ones in the unforced control (not shown). In the 250 years of historical + RCP simulation, however, the anthropogenic trend is large relative to the natural variability, so trends counter to the forced response are rare. This is at the heart of the detection problem: the instrumental record is gradually becoming long enough to resolve interdecadal variability, but there is already a large and growing anthropogenic component. As ocean time-series data accumulate, understanding of the spectrum of variability will increase, but separating out the anthropogenic component will remain extremely challenging.

Using the 2σ YOE estimated here as a benchmark (Figure 5), the last-zero-crossing for a 20-year trend (LZC_20_) is a conservative estimate of emergence, while LZC_30_ has an early-emergence bias (Figure 6). Whether a particular length scale for this analysis is a conservative criterion depends on the specific data sets employed and can not readily be generalized across fields or emissions scenarios (although in these results there are consistent relationships among LZC_20_, LZC_30_ and YOE for different data fields). In general, the faster the anthropogenic signal grows, the stronger the tendency for the LZC to give early emergence. This is a simple function of the signal-to-noise ratio with a nonlinearly growing signal (d^2^y/dt^2^>0) and a constant amount of noise. A noise-driven countertrend that exceeds the forced component is much more likely to occur early in the experiment, when dy/dt is small. Similar logic explains why the criterion becomes more conservative with redder noise. The greater the probability that a trend will occur over a period of e.g. 30 years, the more likely it is that a short-term trend counter to the anthropogenically forced trend will occur in the forced simulation after the ‘true’ emergence point has been passed. For the simulations considered here, the appropriate period appears to be about 25 years (Figure 6).

This analysis - using climate model simulations with future emissions scenarios - obviously benefits from hindsight that can never be available in ocean observations. If a 30 year secular trend is observed, and in the 31st year it does not reverse, nor in the 32nd, the observer is inclined to believe that a long-term trend is present. It is not the presence of a positive trend that is diagnostic, but the absence of counter-trends over shorter periods. Because in observations we lack the hindsight available in models, it is impossible to say with certainty how long a time since LZC is required to diagnose emergence, but model simulations can help us to estimate the probability of a counter-trend emerging in the future. In most locations it is unlikely that counter-trends will ever cease for periods less than about 15 years, even under very strong anthropogenic forcing, although the statistics are likely to be a latitude dependent [27]. For the stations and data fields examined here, LZC is a fairly reliable diagnostic of emergence for a period between 20 and 30 years, with the former being too conservative and the latter giving many false positives (Figure 6).

These results suggest that it will be difficult to detect an anthropogenic trend in localized ocean time-series data with confidence. The existence of a trend on timescales longer than interdecadal does not necessarily imply that it is anthropogenic, but as time series extend beyond the interdecadal range opportunities for detection and attribution will arise. Ultimately the case for anthropogenic forcing will have to rest on a mechanistic understanding of the underlying processes [33]. It has only recently become possible to conduct an analysis of the kind presented here even with models, and the models will continue to improve. At the same time, ocean time series will be extended with modern methods that were novel when the time series began but are now mature and operationalized. Increased computational power also makes possible high resolution regional models that can aid in the interpretation of observations and elucidation of mechanisms.
